# Indoor environmental quality in WELL-certified and LEED-certified buildings

**DOI:** 10.1038/s41598-024-65768-w

**Published:** 2024-07-02

**Authors:** Michael G. Kent, Thomas Parkinson, Stefano Schiavon

**Affiliations:** 1 School of Business, Singapore University of Social Sciences, Singapore, Singapore; 2 Berkeley Education Alliance for Research in Singapore, Singapore, Singapore; 3 School of Architecture, Design and Planning, The University of Sydney, Sydney, NSW Australia; 4 Center for the Built Environment, University of California, Berkeley, CA USA

**Keywords:** Psychology and behaviour, Sustainability

## Abstract

International building certification systems, such as the WELL and Leadership in Energy and Environmental Design (LEED) standards, play a pivotal role in the design of healthy and sustainable buildings. While LEED adopts a holistic approach to designing healthy and sustainable buildings, the WELL standard has a strong emphasis on human health, comfort, and well-being. Although prior research has revealed inconsistent results for occupant satisfaction in office buildings with WELL certification compared to buildings without WELL certification, or are certified using another certification system (e.g., LEED), most of these comparisons tend to lack methodological rigor. This study used a statistical procedure to match and compare 1634 occupant surveys from LEED-certified buildings to 1634 surveys from WELL-certified buildings. Six important architectural and experiential parameters were matched, masking their influence on the outcome. Overall building and workspace satisfaction was high in both WELL-certified buildings (94% and 87%) and LEED-certified (73% and 71%). We found that there is a 39% higher probability of finding occupants who are more satisfied in WELL-certified buildings compared to LEED-certified buildings, indicating occupant satisfaction is higher in buildings with WELL certification. Although we were unable to pinpoint the reason for higher occupant satisfaction in WELL-certified buildings, the results consistently showed that perceived indoor environmental quality was enhanced across all parameters except for the amount of space.

## Introduction

Performance certification standards provide holistic frameworks aimed at designing and operating healthy and sustainable buildings^1^. The premise of these standards is for buildings to accumulate points by meeting specific requirements (e.g., indoor environmental quality, materials and resources, energy performance, etc.). The more points accrued, the higher the building’s rating and the implied performance or quality. Two prominent certification standards are WELL^2^ and Leadership in Energy and Environmental Design (LEED)^3^. Both WELL^4^ and LEED^5^ comprise five certification levels: not certified (< 40 points), bronze/certified (40–49 points), silver (50–59 points), gold (60–79 points), and platinum (> 80 points). Both standards originate from the United States but can be annexed into international applications. LEED has achieved over 100000 certified projects in over 180 countries^6^, while WELL has reached ~ 74000 locations across 136 countries^7^.

Research has questioned whether occupant satisfaction is higher in certified buildings than in their non-certified counterparts^8^. This is typically evaluated through surveys of occupant satisfaction with several important indoor environmental quality (IEQ) parameters such as thermal, air quality, sound, and lighting^9,10^. Studies tend to find that LEED-certified buildings do not outperform non-certified buildings across most IEQ parameters^11,12^. This may be because most certification standards focus on building sustainability and energy performance, with relatively less emphasis placed on IEQ^13^. Similarly, no evidence of increased satisfaction was found when a specific LEED credit had been achieved^1^.

Similar comparisons have been made for other performance certification standards around the world. The Building Research Establishment Environmental Assessment Method (BREEAM) is the common performance certification standard in the United Kingdom. Occupants had lower satisfaction with some IEQ parameters (e.g., privacy, temperature, air quality, and noise) in BREEAM-certified buildings compared to non-certified buildings^13^. In contrast, a comparison of Singapore’s Green Mark certification standard reported consistently higher occupant satisfaction in certified buildings compared to non-certified buildings^14^. This improvement in occupant satisfaction was seen in buildings that had originally received Green Mark certification upon construction, as well as buildings that were later refurbished to achieve Green Mark certification^15^.

The WELL standard is considered a unique certification system compared to other certification standards. While approximately 10% of the total credits in LEED are dedicated to IEQ and other credits target important sustainability criteria, WELL exclusively advocates human-centered building design criteria^2^. Despite the difference in scope, similar inconsistencies in comparisons between certified and non-certified buildings are found in the research literature. Most occupants (55%) of renovated certified buildings (i.e., WELL, BREEAM, and/or LEED) reported no notable difference in occupant satisfaction after relocating into the newly refurbished spaces^16^. Another study also reported mixed results, with higher occupant satisfaction in WELL-certified buildings for spatial and thermal comfort, noise privacy, and personal comfort, but lower satisfaction for visual comfort and connection to the outside^17^. The mixed findings from these studies may be attributed to the types of buildings being compared to those with WELL certification. Some of those buildings were already certified by other standards like BREEAM^16^, and the National Australian Built Environment Rating System or Green Star^17^. In other words, the non-WELL certified cases may already be high-performance buildings in many regards. Another study^18^ compared post-occupancy surveys before and after buildings underwent WELL certification. This study found that occupant satisfaction, and a wider set of parameters such as perceived mental and physical health, and productivity, all appeared to show some improvement following WELL certification. Another study that compared WELL-certified and non-WELL-certified buildings showed marginal improvements to only ‘connection to the outside’ and ‘access to sunlight’^19^.

A challenge when comparing certified and non-WELL certified buildings (e.g., WELL vs. non-WELL, whereby the latter is certified by another system), or buildings with different certification systems (e.g., WELL vs. LEED), is how IEQ parameters are compared. Some studies^16–18^ compare the same building pre- and post-certification, which entails renovating an existing building to meet predefined, and optional optimization, credits to achieve certification. The advantage of this approach is that some features (e.g., architectural dimensions and occupant demographics) will remain relatively constant. However, the inability to conceal interventions generated during the certification process, or masking the presence of confounding factors, are significant drawbacks when attempting to verify evaluations in pre- and post-certification order. Independently comparing different buildings with different occupants, different certification systems, or certified and non-certified buildings, avoids these issues^11,15,19^, but on the other hand, makes it difficult to ensure that other potentially influential features (e.g., proximity to a window or time spent inside the building) are comparable.

Here, we compared two world-leading international certification systems: the WELL standard which focuses on human health, comfort, and well-being, and the LEED standard which champions both building sustainability and IEQ. Although the WELL and LEED standards are separate certification systems, the two standards work collaboratively, and sometimes buildings achieve both certification systems (i.e., dual certification) to obtain their collective benefits. We used a statistical matching technique to maximize the similarity between independent samples from the LEED-certified and WELL-certified buildings. Non-certified buildings were not considered as the presence of both LEED and WELL certification systems has been growing worldwide^6,20^.

### Post-occupancy survey

We used occupant responses to 16 different survey items from the CBE Occupant Survey database^9^. The database had 20 different WELL-certified office buildings with 1634 responses and 49 LEED-certified office buildings with 7152 responses. We used the MatchIt function^21^ embedded in the statistical software R (version 4.1.0) to match 1634 responses from 44 LEED-certified buildings to the 1634 responses from the WELL-certified buildings. The matching was based on six parameters available in the CBE Occupant Survey database, with the resulting samples being summarized in Table 1. More details about this approach are described in the Methods section below.

**Table 1:** Distribution of post-occupant responses for the matched dataset for WELL-certified and LEED-certified building features.

Variable	Response	Frequency (*n*)	Percentage (%)			*χ*^*2*^(df)^NHST^	Effect size
		WELL	LEED	WELL	LEED		
Certification	WELL or LEED	1634	1634	50	50	0.00(1) n.s	0.00 _negligible_
Award level	Silver	247	330	15	20	18.32(2)*	0.07 _negligible_
	Gold	248	272	15	17		
	Platinum	1139	1032	70	63		
Years in building	Less than 1 year	300	459	18	28	58.99(3)*	0.13 _negligible_
	1–2 years	672	676	41	41		
	3–5 years	294	197	18	12		
	More than 5 years	368	302	23	19		
Time at workspace	Less than 3 months	119	161	7	10	14.16(3)*	0.06 _negligible_
	3–5 months	154	196	9	12		
	7–12 months	381	366	23	22		
	More than 1 year	980	911	60	56		
Type of workspace	Enclosed office	264	235	14	16	2.63(2) n.s	0.02 _negligible_
	Open plan office	1324	1344	82	81		
	Other	46	55	3	3		
Near a window	Yes	1329	1337	81	82	0.01(1) n.s	0.00 _negligible_
	No	305	297	19	18		

Differences were statistically significant for award level, years in building, and time at workspace (*χ*^*2*^ tests, *p* ≤ 0.005), but all the differences were ‘negligible’ in size (ρ < 0.20). The median floor height (M_dn_ = 3) was the same for the WELL-certified and LEED-certified buildings (Mann–Whitney U test, *p* = 0.05, ρ = − 0.03 (‘negligible’ difference)).

*Indicates statistically significant differences at *p* ≤ 0.005 for the null hypothesis significant test (NHST) and n.s. indicates differences that were not statistically significant^22^.

Negligible effect sizes: Cramer’s φ or V =  < 0.20^23^.

**Figure 1:**
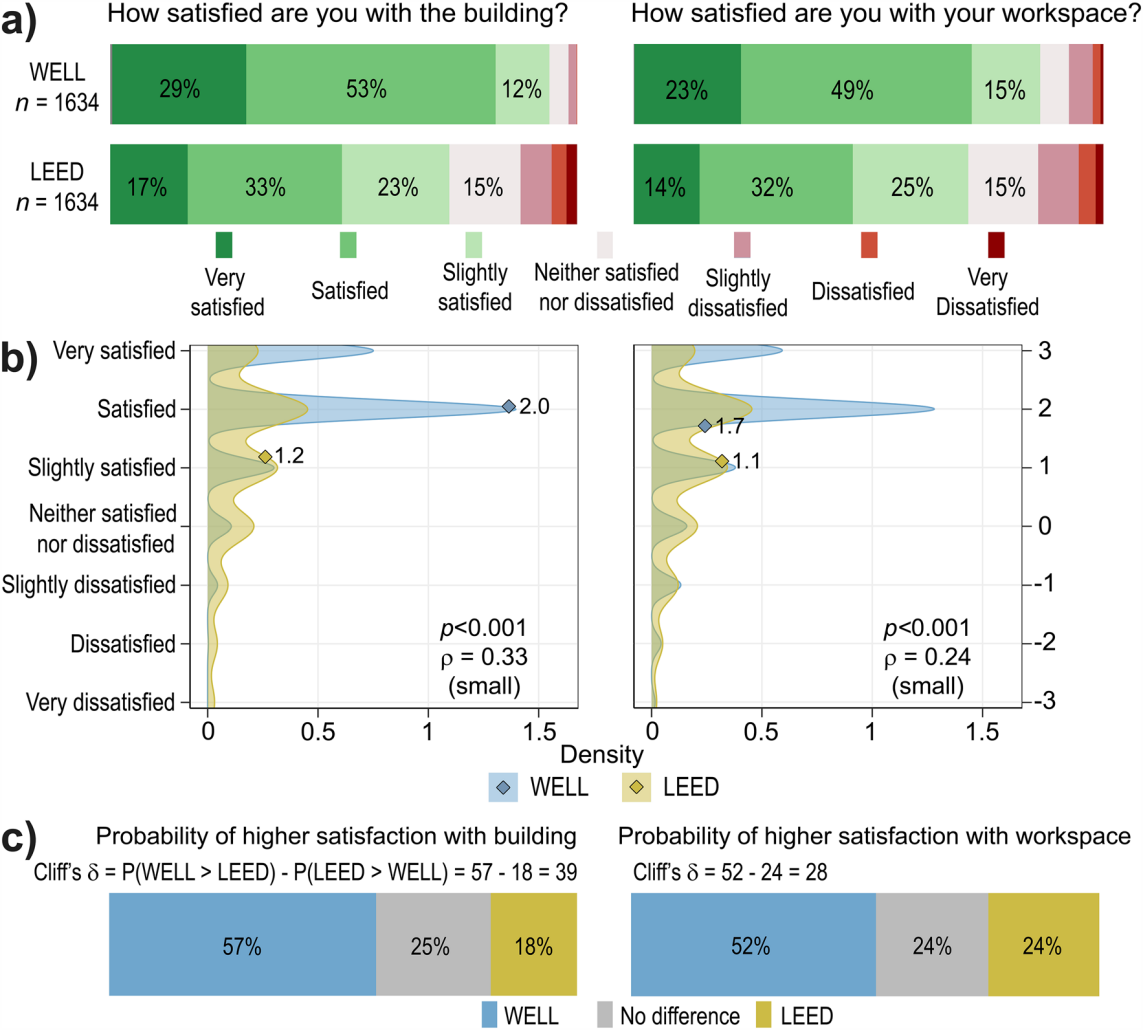
Statistical analyses that compare the 3268 matched responses from WELL (n = 1634) and LEED (n = 1634) certified buildings for satisfaction with the overall building (left) and workspace (right). (**a**) Distributions of occupant satisfaction. The numerical values indicate the percentage of occupant responses, indicating higher satisfaction in WELL-certified buildings for both overall building and workspace. (**b**) Density plots show the concentration of responses on the 7-point Likert satisfaction scale. The diamonds indicate the mean values based on the numerically coded values that correspond to the survey’s scale. The mean values for both satisfaction items are higher in WELL-certified buildings. The Mann–Whitney U test shows that the differences in occupant satisfaction are statistically significant (*p* ≤ 0.005)^22^ and the effect size is practically significant (ρ > 0.20) for the overall building and for the workspace^23^. (**c**) Probabilities of higher satisfaction occurring in WELL-certified or LEED-certified buildings, or there being no difference, when randomly selecting two responses from both certification groups. We resampled from the original dataset to generate 1000 unique responses for the WELL and LEED groups. The Cliff’s δ^24^ shows there are higher probabilities of selecting an occupant response associated with higher satisfaction in a WELL than in a LEED-certified building.

Figure 1 compares occupant satisfaction with the overall building and workspace for the matched sets of WELL-certified and LEED-certified buildings. The results in Fig. 1a show that the percentage of satisfied responses is higher for WELL-certified buildings for both satisfaction with the building and workspace. This result is supported by the density plots in Fig. 1b, which both show that more occupants had a higher level of satisfaction in WELL-certified buildings. General satisfaction with the building and workspace was high in both WELL-certified (94% and 87%) and LEED-certified (73% and 71%) buildings. Increased probability of higher satisfaction occurs for both the building (57%) and workspace (52%) for WELL-certified buildings when randomly selecting an occupant response (see Fig. 1c). There is a 39% higher probability of finding an occupant that is satisfied with the building overall in a WELL-certified building compared to a LEED-certified building.

**Figure 2:**
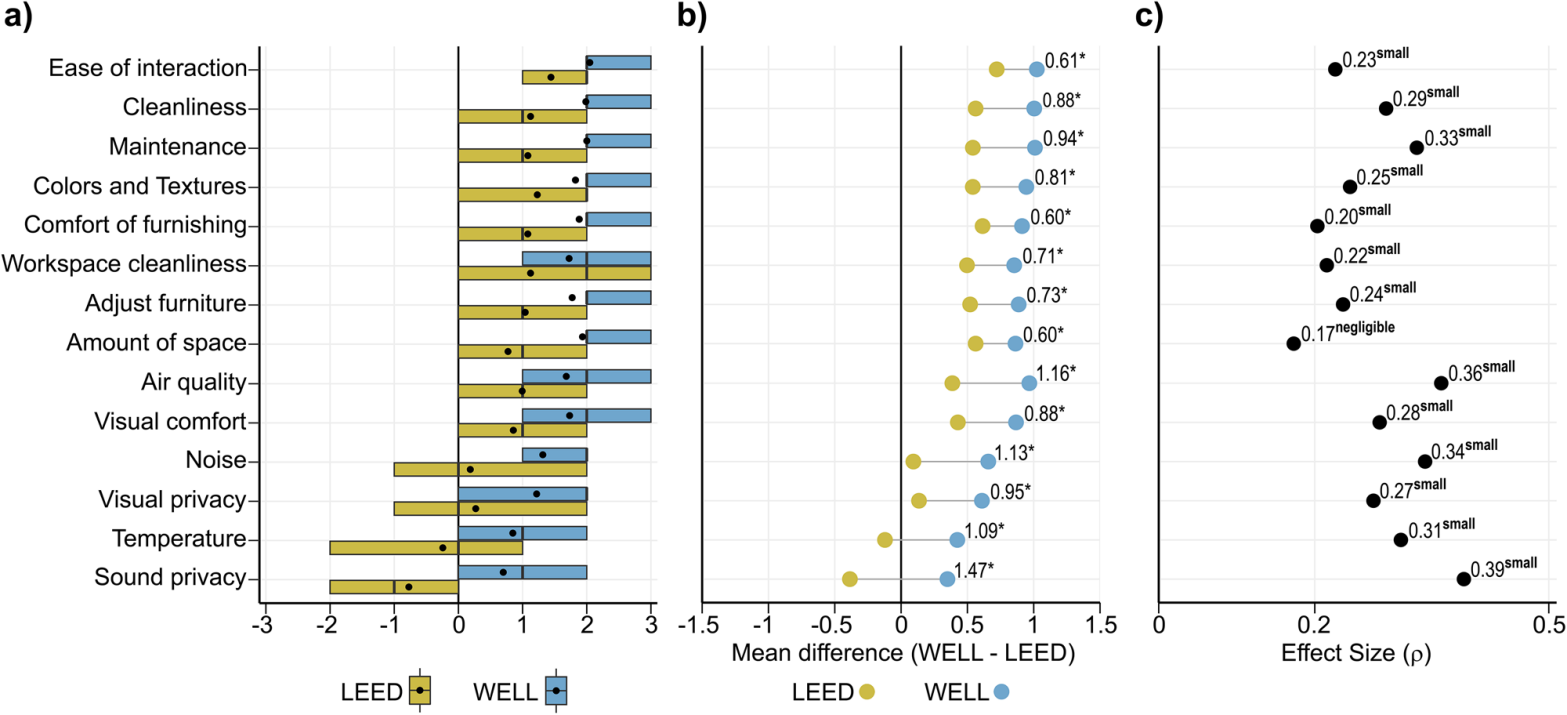
Plots comparing satisfaction with 14 IEQ parameters in WELL-certified and LEED-certified buildings. (**a**) Inverted boxplots comparing the 25th, 50th (median), and 75th percentiles. The circles are the mean values. The IEQ parameters have been ordered from increasing (‘ease of interaction’) to decreasing satisfaction (‘sound privacy’). Occupant satisfaction is consistently higher in WELL-certified buildings for every IEQ parameter. (**b**) The mean difference between WELL-certified and LEED-certified buildings and the associated statistical significance according to the Mann–Whitney U test. (*) indicates that every comparison was statistically significant (*p* ≤ 0.005). (**c**) Effect sizes (ρ) for each comparison. The differences are ‘small’ (0.20 ≤ ρ < 0.50) for 13 IEQ parameters and ‘negligible’ (ρ < 0.20) for the parameter ‘amount of space’. The largest effect size was for ‘sound privacy’.

Figure 2 presents the comparison of satisfaction with 14 other IEQ parameters from the CBE Occupant Survey. Figure 2a shows higher occupant satisfaction across all items in WELL-certified buildings. The differences between WELL-certified and LEED-certified buildings were all statistically significant (Fig. 2b). The effect sizes in Fig. 2c showed that 13 comparisons across WELL-certified and LEED-certified buildings were ‘small’. Satisfaction with the ‘amount of space’ was the only ‘negligible’ effect size. Sound and visual privacy, temperature, and noise had the lowest satisfaction, which has been shown in previous work^10,24,25^. The largest difference in satisfaction and effect size was for ‘sound privacy’, which was unexpected given that there was no statistically significant difference in the type of workspace (i.e., enclosed, or open-plan office) between the two samples.

Our analysis of the CBE Occupant Survey Database showed that occupants of WELL-certified buildings tend to have higher satisfaction with their office environment than their counterparts in LEED-certified buildings, similar to findings shown in past research^18^. However, our current analysis was supported by a more robust statistical approach^21^, enabling us to fairly compare WELL-certified and LEED-certified buildings. Even though higher satisfaction was found in WELL-certified buildings, satisfaction in LEED-certified buildings to the building, workspace, and most individual IEQ parameters was still relatively high.

Interestingly, several buildings in our database have achieved both WELL and LEED certification. We excluded dual-certified buildings from our analysis to enable a fair comparison between WELL and LEED-certified buildings. However, building owners and managers may be aware of the sustainability and human-centered benefits of both internationally leading certification systems since they have already achieved dual certification. It is difficult to pinpoint the precise reasons for higher satisfaction in WELL-certified buildings. Given that WELL is touted as a human-centered building design standard^2^, a plausible reason could be that the accreditation process itself directly influenced design enhancements leading to improved occupant satisfaction. These pursued credits may not be available in the LEED certification system, which contains fewer criteria targeting human comfort, health, and well-being^3^. For example, WELL version 2^2^ offers ‘sound mapping’ as a precondition that must be met to achieve certification. This requires designers to include a quiet zone for privacy and an acoustic design plan that could include solutions that safeguard speech privacy. Given the relative importance of acoustic privacy in determining overall satisfaction with the workspace^10^, these design decisions could lead to meaningful improvements in WELL-certified buildings. To obtain higher satisfaction in LEED-certified buildings, an argument could be proposed to integrate some credits unique to the WELL standard into the LEED standard. Compliance pathways for some credits that are similar (e.g., acoustic performance) in both standards have recently been proposed^25^. Although it remains unclear which or how many WELL credits need to be included in the LEED certification process, targeting individual IEQ parameters with the largest differences in satisfaction (Fig. 2c) could help bridge the difference in building and workspace satisfaction across the two certification systems. This may be preferable when dual certification is not feasible due to certain constraints (e.g., time or budget), preventing buildings from achieving the benefits possible through LEED and WELL certification.

The other possible reason for higher satisfaction in WELL-certified buildings could be the age of the building or workspace. Occupants of newer spaces tend to be more satisfied than those who have been in the workspace longer^12,26^. Unfortunately, we do not know the age of all the buildings in our sample, but the WELL-certified buildings were likely newer than their LEED counterparts. Ideally, the matching process would include building attributes and occupant parameters as control variables.

## Methods

Using post-occupancy surveys to collect subjective feedback from occupants is considered a ‘basic’ level of IEQ performance evaluation in buildings^27^. This is typically measured by surveys designed to collect information about occupants’ perceptions of the indoor environment and workspace layout. We used the CBE Occupant Survey, which is one of the longest-running post-occupancy evaluation tools^9^. All analyses were performed in R (version 4.1.0) and RStudio (version 1.4), along with additional packages used as described in the following sections.

### CBE occupant survey

The survey was developed and administered by the Center for the Built Environment (CBE) at the University of California, Berkeley. It is an online post-occupancy evaluation tool designed to evaluate indoor satisfaction with the building and workplace^28^. Survey respondents are emailed a link to the survey which requires approximately 15 min to complete. The total dataset contains approximately 90000 responses from almost 900 buildings since 2001^9^.

The survey contains questions about demographics (e.g., age and gender), experience inside the space (e.g., years in building, time at workspace, office layout, proximity to a window etc.), and satisfaction with different aspects of the indoor environment, including air quality, amount of light, amount of space, cleanliness, colors and textures, comfort of furnishings, ease of interaction, furniture adjustment, noise, sound privacy, thermal comfort, visual comfort, visual privacy, and workspace cleanliness. Two questions measure overall satisfaction with the building and personal workspace. We used occupant responses from buildings that had been WELL-certified and LEED-certified. Satisfaction items are evaluated using a 7-point Likert scale, ranging from ‘Very Dissatisfied’ (− 3) to ‘Very Satisfied’ (+ 3), which are balanced across an indifference point (0).

### Data matching

We used 1634 responses from 20 different WELL-certified buildings in the CBE dataset. There were more LEED-certified buildings in the CBE dataset, with 7152 responses from 49 buildings. A simple data pre-processing approach enabled us to closely match a set of control variables to ensure that the response variables were independent of confounding factors^29^. We used the MatchIt function^20^ in the R MatchIt package to create two datasets of equal size.

Data pre-processing was one of the most important processes to ensure the independent WELL and LEED datasets contained the same variables required for matching. This meant that the same IEQ parameters, and other variables (e.g., award level) that were matched, appeared in the same order along the columns in both datasets to enable them to be successfully harmonized. The resulting subset comprised 1634 of the closest responses from the larger LEED datasets to the WELL dataset. The LEED dataset was matched according to the following variables: award level, years in building, time in workspace, type of workspace, proximity to a window, and floor height. The “Tidyverse” suite of packages^30^ was used for data wrangling, curation, and plotting.

Occupant satisfaction with workspaces is known to vary due to habitual effects related to the time spent inside the building and workspace^12,31^, the type of workspace (e.g., enclosed or open-plan office)^10,24,25^, floor elevation^32^, and proximity to the window^33^. Floor elevation and window proximity influence how the view out of the building is perceived but can also affect other aspects, such as thermal and visual comfort^34^. Although no evidence has been found to suggest occupant satisfaction increased according to the total number of IEQ points earned in LEED-certified buildings^1^, it is unclear whether this also occurs in WELL-certified buildings. Therefore, the LEED dataset was also matched according to the certification level.

### Limitations

There are several limitations when using post-occupancy evaluations. While we masked the potential influence of several parameters on the outcome using the statistical matching approach, there remain other architectural features within the buildings or personal factors that affect occupant satisfaction. Other dimensions (e.g., age of the building or job satisfaction) were not controlled variables in our matching procedure but are known to influence workspace satisfaction. However, including more matched variables may unintentionally increase the variability of other matched variables. It is not known which control variables are most important for this comparison.

Another caveat is that occupant surveys are unable to characterize every aspect of the workspace experience or indoor environment. Satisfaction is one of many dimensions perceived by building occupants. Dissatisfaction with temperature could lead to thermal discomfort (e.g., too hot) but dissatisfaction with privacy that hinders the occupants’ ability to perform office tasks may not necessarily cause any physical discomfort^10^. Therefore, it is unclear whether the results reported here for satisfaction would be seen for other dimensions such as comfort. As a result, we are unable to understand exactly why higher satisfaction was found in WELL-certified buildings.

### Ethics statement

The CBE occupant survey was approved by the Internal Review Board at the University of California, Berkeley: Grant number IRB-2010-05-1550. All responses collected were anonymous and informed consent was obtained from each surveyed participant. All research was conducted in accordance with the principles outlined in the Belmont Report.

## Data Availability

The CBE Occupant Survey database generated and/or analyzed during the current study is not publicly available to comply with the approved IRB protocol at the University of California, Berkeley (IRB-2010-05-1550). Data summaries related to this paper may be requested from the corresponding author.
